# Isolation and Molecular Characterization of *Corynebacterium pseudotuberculosis*: Association with Proinflammatory Cytokines in Caseous Lymphadenitis Pyogranulomas

**DOI:** 10.3390/ani13020296

**Published:** 2023-01-14

**Authors:** Helmy A. Torky, Hebatallah M. Saad, Samy A. Khaliel, Asmaa T. Kassih, Jean-Marc Sabatier, Gaber El-Saber Batiha, Helal F. Hetta, Eman M. Elghazaly, Michel De Waard

**Affiliations:** 1 Department of Microbiology, Faculty of Veterinary Medicine, Alexandria University, Abees, Alexandria 21523, Egypt; 2 Department of Pathology, Faculty of Veterinary Medicine, Matrouh University, Marsa Matruh 51744, Egypt; 3 Institut de Neurophysiopathologie (INP), CNRS UMR 7051, Faculté des Sciences Médicales et Paramédicales, Aix-Marseille Université, 27 Bd Jean Moulin, F-13005 Marseille, France; 4 Department of Pharmacology and Therapeutics, Faculty of Veterinary Medicine, Damanhour University, Damanhour 22511, Egypt; 5 Department of Medical Microbiology and Immunology, Faculty of Medicine, Assiut University, Assiut 71515, Egypt; 6 Department of Microbiology, Faculty of Veterinary Medicine, Matrouh University, Marsa Matruh 51744, Egypt; 7 Smartox Biotechnology, 6 Rue des Platanes, F-38120 Saint-Egrève, France; 8 L’institut Du Thorax, INSERM, CNRS, UNIV NANTES, F-44007 Nantes, France; 9 LabEx «Ion Channels, Science & Therapeutics», Université de Nice Sophia-Antipolis, F-06560 Valbonne, France

**Keywords:** *Corynebacterium pseudotuberculosis*, ERIC-PCR, NF-κB/p65, IL1β, TNF, caseous lymphadenitis

## Abstract

**Simple Summary:**

Caseous lymphadenitis (CLA) is caused by *Corynebacterium pseudotuberculosis (C. pseudotuberculosis)* and is considered one of the most serious infectious diseases in small ruminants with poor efficacy of treatment. In this study, *C. pseudotuberculosis* was isolated from 120 abscessed lymph nodes (LNs) and organs at the Matrouh abattoir in Egypt, confirmed by PCR and by intraperitoneal injection of male Guinea pigs, and then characterized for antimicrobial susceptibility and its genetic-relatedness by enterobacterial repetitive intergenic consensus polymerase chain reaction (ERIC-PCR). Gross examination of affected LNs and organs revealed marked enlargement with caseated green pus surrounded by a dense fibrous capsule. Cross section of some LNs exhibited an onion ring-like appearance, which is a pathognomonic feature of CLA. In immunohistochemical staining, IL1β is a more crucial proinflammatory cytokine than TNF in the regulation of *C. pseudotuberculosis* infection, which is accompanied by marked NF-κB expression. This study aids in the epidemiological investigation and preliminary etiological analysis in the general development of ERIC-PCR for *C. pseudotuberculosis* genotyping and characterization along with susceptibility to antibiotics to select an appropriate treatment.

**Abstract:**

*Corynebacterium pseudotuberculosis (C. pseudotuberculosis)* is a causative agent of numerous chronic diseases, including caseous lymphadenitis (CLA) in sheep and goats, which has a zoonotic potential in humans in addition to a poor therapeutic response. In this study, out of 120 collected samples, only 12 (10%) were positive for *C. pseudotuberculosis* by PCR and by intraperitoneal injection of male Guinea pigs and then characterized for antimicrobial susceptibility and its genetic-relatedness by enterobacterial repetitive intergenic consensus polymerase chain reaction (ERIC-PCR), which showed 2–4 bands ranging from 100 to 3000 bp that can be clustered into four clusters (C1–C4). Despite the serotype biovar 1 only infecting sheep and goats, ERIC–PCR reveals intra-subtyping variation. Examination of affected LNs and organs revealed marked enlargement with either thick creamy green pus or multiple abscesses of variable sizes with a central caseated core surrounded by dense fibrous capsule. A histopathological examination revealed a central necrotic core surrounded by a peripheral mantle of mononuclear cells and a fibrous capsule. Positive immune expression of nuclear factor kappa B (NF-κB/p65) and interleukin-1β (IL-1β) and negative expression of tumor necrosis factor (TNF) in CLA is the first report to our knowledge. Conclusion: In CLA pyogranulomas, IL1β is a more crucial proinflammatory cytokine than TNF in the regulation of *C. pseudotuberculosis* infection, which is accompanied by marked NF-κB immunoexpression. Therefore, the NF-κB/p65 signaling pathway is involved in the activation of IL1β, and additional immunohistochemical studies are required to determine the various roles of NF-κB/p65 in the inflammatory response within CLA pyogranulomas to control this pathogen.

## 1. Introduction

*Corynebacterium pseudotuberculosis (C. pseudotuberculosis)* is the causative agent of several chronic conditions that affect sheep, goats, horses, cattle, and occasionally humans [1,2,3,4] but sheep and goats are most susceptible to this organism which causes caseous lymphadenitis (CLA).

Unlike other *Corynebacterium* species, *C. pseudotuberculosis* is a facultative intracellular bacterium, a non-sporulated, non-capsulated, non-motile Gram-positive pleomorphic bacterium [5,6]. It causes orchitis and inflammation of the scrotal of a male Guinea pig when inoculated intraperitoneally (Strauss reaction), to differentia it from another *Corynebacterium* species [7]. It has two major virulence factors, an invasive phospholipase-D (PLD) exotoxin and a mycolic acid-rich cell wall [8,9], resulting in a poor response to therapeutics and difficult disease eradication [1].

*C. pseudotuberculosis* biovar 1 (serotype 1) causes CLA and the disease manifests in two forms: external (superficial or cutaneous), characterized by abscess formation in superficial lymph nodes (LNs) or subcutaneous tissue; and visceral, characterized by abscess formation in internal LNs (primarily mediastinal LNs) and other organs, particularly lungs. In addition, these lesions can be found in numerous organs, including the liver, kidneys, and mammary glands (MGs), but only rarely in the heart, testes, scrotum, uterus, joints, brain, and spinal cord. Abscess in the early stage contains greenish-yellow pus that develops into a lamellated lesion resembling an onion ring, which is pathognomonic for CLA in small ruminants [10]. A postmortem examination of a few animals without obvious clinical symptoms revealed their infection with the bacterium [8].

Clinical diagnosis of caseous lymphadenitis is straightforward if superficial abscesses are present. However, it is important to differentiate caseous lymphadenitis from the other causes of abscess disease. Moreover, serological techniques such as ELISA were developed to identify infected animals because it is difficult to detect live animals with small abscesses or internal abscesses. A definite diagnosis requires a laboratory bacteriological examination of the pus and identification of *C. pseudotuberculosis* via microscopic examination (thick Gram-positive bacilli), culture characteristics (on sheep blood agar media, *C. pseudotuberculosis* appears as round, whitish, brilliant, and ‘slippery’ colonies, surrounded by a slight hemolysis zone), biochemical reactions (Catalase-positive, Urease-positive, Nitrate reductase-negative, indole-negative, H2S-negative, ferment glucose, maltose, mannose, fructose, and glycogen), serological identification by ELISA, and molecular identifications using PCR and sequencing [8,9,10]. Treatment and control measures include surgical treatment of abscesses and culling of severely infected animals, proper antibiotic treatment based on results of antibiotic susceptibility testing, vaccination, and selection of resistant breeds [8,9,10].

Enterobacterial repetitive intergenic consensus polymerase chain reaction (ERIC-PCR) is one of the DNA-based methods used to investigate the genetic diversity of Gram-positive [11] and Gram-negative bacteria [12]. This PCR method’s ability to amplify minute amounts of microbial DNA sequences has made it a potent molecular tool [13,14], de Sa Guimaraes et al. utilized ERIC-PCR for subtyping *C. pseudotuberculosis* [15].

Nuclear factor kappa-light-chain-enhancer of activated B cells (NF-B/p65) is essential for the expression of proinflammatory cytokine genes, such as interleukin-1β (IL-1β) and tumor necrosis factor (TNF), in response to lipopolysaccharide or microbial stimuli [16,17]. IL-1β is regarded as one of the most important proinflammatory cytokines in acute inflammatory responses and plays multiple roles in the defense against bacterial infections. The primary secretion mechanism of IL-1β in CLA pyogranulomas is unknown but two signaling pathways that may be incorporated into the IL-1β production: signal I cause NF-κB-dependent expression of both IL-1β precursor (pro-IL-1β) and inflammasome after pattern-recognition receptors (PRRs), and signal II provokes assembly and inflammasome activation. Inflammasome activation leads to cleavage of the pro-IL-1β into mature IL-1β by active caspase-1 [18,19].

This study aimed to elucidate the epidemiological investigation and preliminary etiological analysis in the general development of ERIC-PCR to detect *C. pseudotuberculosis* genotype, characterize their susceptibility to antibacterial to select suitable antibacterial for treatment, and detect pathological changes and the immunohistochemical expression of NF-κB/p65, IL1β, and TNF within CLA granulomas.

## 2. Material and Methods

### 2.1. Media and Culture Conditions

A total of 120 samples were collected from closed abscessed lymph nodes and organs of slaughtered sheep and goats in the slaughterhouse of Matrouh Governorate. Swabs from incised organs and aspirates from an abscess using syringes were immersed into peptone water broth and transferred in an ice box to the laboratory. Samples were subjected to bacteriological examination as soon as possible. The swabs were inoculated onto brain heart infusion agar (Oxoid) supplemented with 200 mg of fosfomycin (Sigma,) and 4 mg/liter of nalidixic acid and incubated at 37 °C for 48 h [4].

### 2.2. Identification of C. pseudotuberculosis

Biochemical identification of *C. pseudotuberculosis* [8] by colonial characteristics, microscopic examination, biochemical identification (catalase test, urease test, Nitrate reduction test, gelatin liquefaction, and negative in lactose, trehalose, and motility test [20]), and measurement of hemolytic activity by modified the Christie–Atkins–Munch-Peterson (CAMP) test [21].

Pathogenicity testing was performed by injecting male Guinea pigs intraperitoneally and isolating organisms from orchitis lesions and other internal lesions [22].

Genotypic identification of *C. pseudotuberculosis* was performed by PCR for detection of the 16s RNA. Primers and PCR conditions to confirm the identification of *C. pseudotuberculosis* isolates [23] are shown in Table 1.

### 2.3. Antimicrobial Susceptibility

Based on the Kirby–Bauer (disc diffusion test) method employing a bacterial suspension with turbidity standards of 0.5 McFarland and Muller Hinton agar plates, as well as the recommendation of the national committee for clinical laboratory standards institute, the following was determined: [24] Isolates were classified according to the Table 2 and Table 3 by Magiorakos et al. [25]. Primers and PCR conditions [26] confirm the presence of integron in a strain with resistance to six antibiotic classes as shown in Table 1.

### 2.4. Integron1 Gene Cassette Detection:

The molecular detection of integrin 1 was carried out in Biotechnology Department, Animal Health Research Institute, Dokki, Giza, Egypt as follows.

#### 2.4.1. DNA Extraction

DNA extraction from samples was performed using the QIAamp DNA Mini kit (Qiagen, Germany, GmbH) with modifications from the manufacturer’s recommendations. Briefly, 200 µL of the sample suspension was incubated with 10 µL of proteinase K and 200 µL of lysis buffer at 56 O C for 10 min. After incubation, 200 µL of 100% ethanol was added to the lysate. The sample was then washed and centrifuged following the manufacturer’s recommendations. Nucleic acid was eluted with 100 µL of elution buffer provided in the kit.

#### 2.4.2. Oligonucleotide Primer

Primers were supplied from Metabion (Germany) and are listed in Table 1.

PCR amplification: Primers were utilized in a 25 µL reaction containing 12.5 µL of DreamTaq Green PCR Master Mix (2X) (Thermo Scientific), 1 µL of each primer of 20 pmol concentration, 5.5 µL of water, and 5 µL of DNA template. The reaction was performed in an Applied biosystem 2720 thermal cycler.

#### 2.4.3. Analysis of the PCR Products

The products of PCR were separated by electrophoresis on 1% agarose gel (Applichem, Germany, GmbH) in 1x TBE buffer at room temperature using gradients of 5 V/cm. For gel analysis, 20 µL of the PCR products were loaded in each gel slot. Gene ruler 100 bp DNA ladder (Fermentas, Sigma) was used to determine the fragment sizes. The gel was photographed by a gel documentation system (Alpha Innotech, Biometra) and the data were analyzed through computer software.

### 2.5. ERIC-PCR

According to Versalovic et al. enterobacterial repetitive intergenic consensus polymerase chain reaction (ERIC-PCR) and ERIC1/ERIC2 primers were synthesized and used [27].

ERIC1, 5- ‘‘ATG TAA GCT CCT GGG GAT TCA C-3’’
ERIC-2, 5- ‘‘AAG TAA GTG ACT GGG GTG AGG G-3’’

The DNA was extracted according to Pitcher et al. [28]. Spectrophotometry was used to +determine the DNA’s quality and concentration [29]. The Animal Health Research Institute in Dokki, Giza, Egypt, conducted all molecular characterization.

Subsequently, using 100 bp and 3000 bp, the size of amplified fragments was determined after electrophoresis in a submerged agarose gel (1.5%), as described by Sambrook [30]. Following electrophoresis, we analyzed the banding patterns for each individual isolate. Bands at the same location in all isolates were recorded as 1, and the absence of a particular band was recorded as 0. All data were analyzed using the POPGENE (Version 1.31) program [31]. A dendrogram of dissimilarity was constructed with all the isolates.

### 2.6. Sample Collection for Histopathological Examination

Affected lymph nodes and organs (liver, lung, and MG) from natural CLA-infected sheep and goats with testicles and epididymis from Strauss reaction’s positive Guinea pigs were carefully excised and immediately fixed in 10% neutral buffered formalin. Animals with negative bacterial isolation and PCR results were culled. Positive samples were prepared using the paraffine embedding technique [32] for histopathological examination and stained with H and E.

### 2.7. Evaluation of the Expression of NF-κB, IL1β, and TNF by Immunohistochemistry

The expression of NF-κB, IL1β, and TNF was determined using a standard horseradish peroxidase immunohistochemical technique. Briefly, 4–5 μm deparaffinized sections of LNs, lung, liver, and MG were mounted on positively charged slides and pre-treated with 3% H_2_O_2_ to inhibit endogenous peroxidase activity. Slides were microwaved for 10 min in 10 mM sodium citrate buffer (pH 6.0) to retrieve antigen. DAKO Corporation’s monoclonal antibodies for NF-κB/p65, IL1β, and TNF, DAKO Corp. were applied to slides and incubated overnight at 4 °C in a humid chamber. Then, a secondary biotinylated antibody was applied, followed by 3,3′-diaminobenzidine tetrahydrochloride incubation (DAB). After each step, sections were washed with phosphate buffer saline three times. After dehydration and DPX mounting, sections were stained with diaminobenzidine chromogen solution and counterstained with Mayer’s hematoxylin [33].

## 3. Results

### 3.1. Bacteriological Identification and Antimicrobial Susceptibility

**Figure 1:**
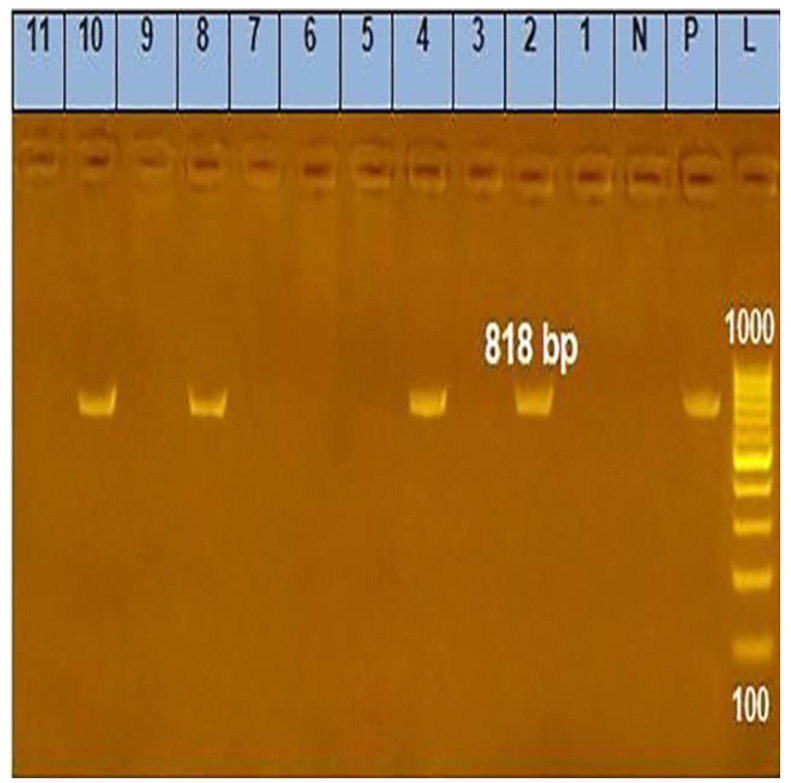
Agarose gel electrophoresis (1.5% and stained with ethidium bromide) showing the amplified *16S rRNA* gene of the isolated *C. pseudotuberculosis*. Lane (L): DNA molecular weight ladder (100 bp ladder); lane (P): positive control for the *C. pseudotuberculosis 16S rRNA* gene; lane (N): negative control for the *C. pseudotuberculosis 16S rRNA* gene; lanes (2, 4, 8, and 10): Positive results for *C. pseudotuberculosis 16S rRNA* gene (specific band at 818 base pairs); lanes (1, 3, 5, 6, 7, 9, and 11): negative results for *C. pseudotuberculosis 16S rRNA*.

Identification of *C. pseudotuberculosis* was performed by microscopic examination showing Gram-positive bacilli, culture characteristics and colonial morphology (on sheep blood agar media, *C. pseudotuberculosis* appears as round, whitish, brilliant, and ‘slippery’ colonies, surrounded by a slight hemolysis zone), and biochemical reactions (Catalase-positive, Urease-positive, Nitrate reductase-negative, indole-negative, H2S-negative, gelatin liquefaction-negative, ferment glucose, maltose, mannose, fructose and glycogen). Based on the phenotypic identification, out of 120 collected samples, only 12 (10%) were positive for *C. pseudotuberculosis* and were confirmed by PCR as depicted in (Figure 1), and by intraperitoneal injection of male Guinea pigs, whereas two did not produce any lesion (83.3% positive in Strauss reaction).

**Figure 2:**
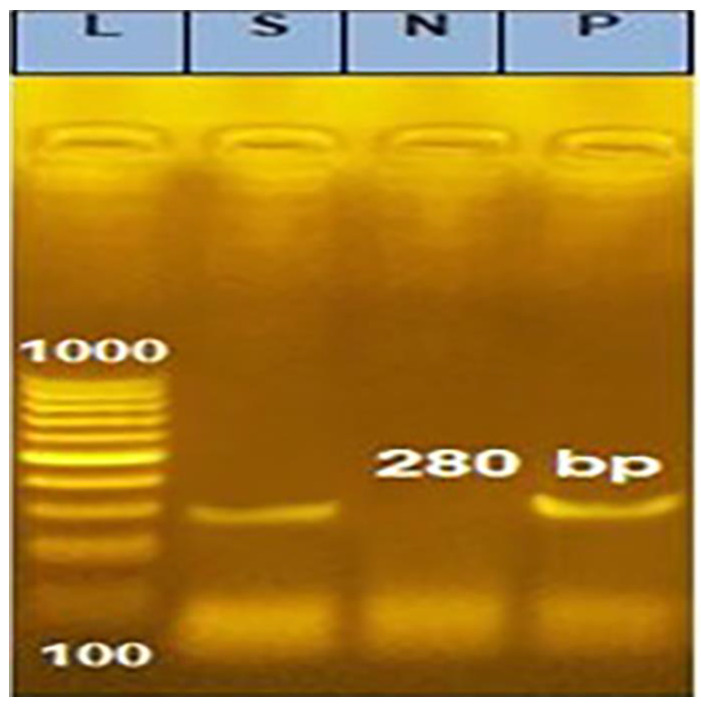
Agarose gel electrophoresis (1.5% and stained with ethidium bromide) for detection of integron gene in *C. pseudotuberculosis* isolate showed resistance to 6 classes of antibiotics. Lane (L): DNA molecular weight ladder (100 bp ladder); lane (S): positive result for integron gene (specific band at 280 bp); lane (N): negative control for integron gene; and lane (P): positive control for integron gene.

As shown in Table 2 and Table 3, 12 *C. pseudotuberculosis* isolates exhibited resistance to various classes of antibiotics. *C. pseudotuberculosis* isolate resistant to six antibiotic classes used in this study was integron gene-positive, as shown in Figure 2.

**Table 2:** Results of antibiotic sensitivity for *C. pseudotuberculosis* strains.

	1	2	3	4	5	6	7	8	9	10	11	12
Erythromycin	R	S	R	R	R	S	R	S	R	R	R	S
Doxycycline	S	S	S	S	S	S	S	S	S	S	S	S
Ciprofloxacin	S	S	S	S	S	S	S	S	S	S	S	S
Azithromycin	S	S	S	R	S	S	S	S	S	S	S	S
Cefaclor	R	S	R	S	R	S	R	R	R	R	R	R
Amoxicillin	R	S	R	S	R	S	R	R	R	R	R	R
Gentamicin	S	S	S	S	R	S	R	S	R	R	S	R
Kanamycin	S	S	S	S	S	S	S	S	R	R	S	S
Mupirocin	S	S	S	R	R	S	R	R	S	R	R	R
Clindamycin	S	S	S	S	S	S	R	S	S	S	S	S
Ampicillin	R	S	R	S	R	S	S	S	R	R	R	S

S: sensitive; R: resistant.

**Table 3:** The antibiotic resistance pattern of the isolated *C. pseudotuberculosis* to antimicrobial classes.

Number of Examined Isolates	Antibiotic Resistance Pattern	Resistance to Antimicrobial Classes
3	CF, AMX, AMP	2 classes
2	MUP, AMX, GEN, CF and AMP	4 classes
1	MUP, AMX, AMP, CF, K, GEN	4 classes
1	GEN, AMX, AMP, MUP, CF	4 classes
1	AMX, CF, MUP	3 classes
1	K, AMX, AMP, CF, GEN	3 classes
1	AMX, AMP, CF, MUP	3 classes
1	AT, MUP	2 classes
1	MUP, CF, E, GEN, AMX, CD	6 classes

CF: cefaclor; AMX: amoxicillin; AMP: ampicillin; MUP: mupirocin; GEN: gentamycin; K: kanamycin; AT: azithromycin; E: erythromycin; CD: clindamycin.

**Figure 3:**
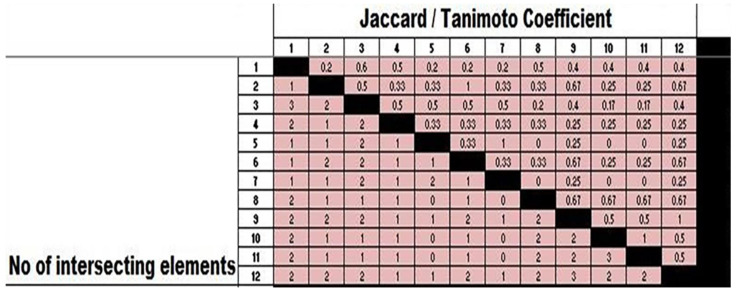
Similarity index (Jaccard/Tanimoto Coefficient and the number of intersecting elements) between 12 isolates of investigated *C. pseudotuberculosis*.

**Figure 4:**
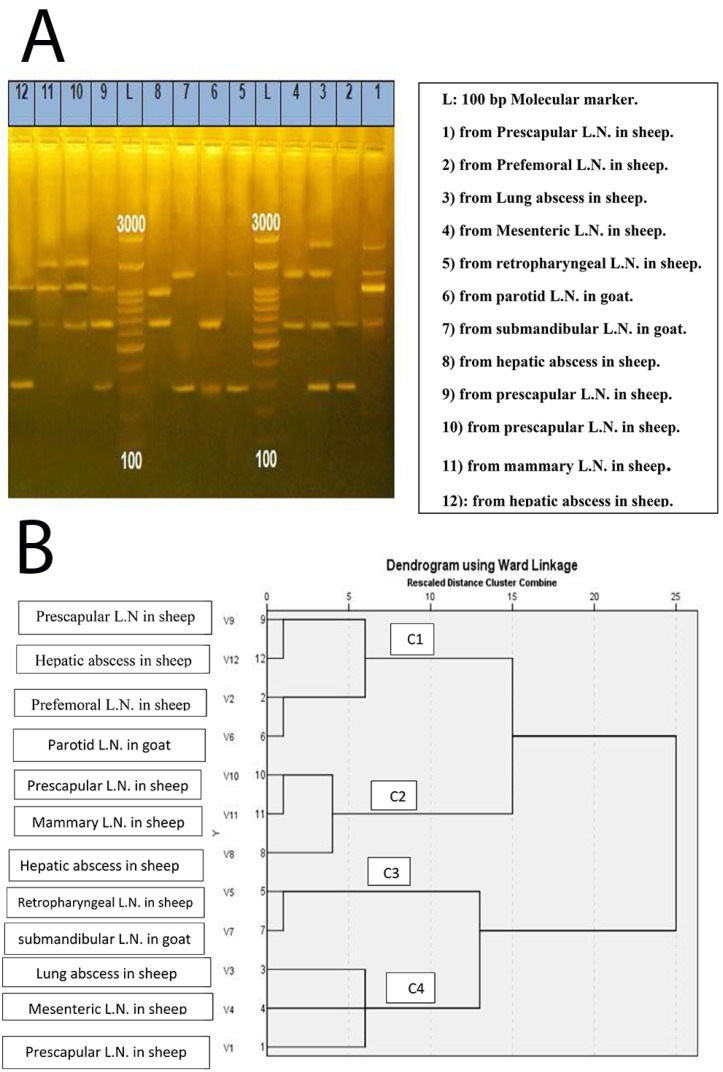
ERIC-PCR of 12 isolates of *C. pseudotuberculosis*. (**A**) ERIC-PCR fingerprinting in an agarose gel containing 1.5% agarose. (**B**) A dendrogram illustrating the relatedness of the isolates.

Isolates 2, 6, 9, and 12 of *C. pseudotuberculosis* were obtained from the prefemoral L.N. in sheep, the parotid L.N. in goats, prescapular L.N. in sheep, and hepatic abscess in sheep were clustered with a 7% dissimilarity in the same cluster (C1). Similarly, isolates No. 8, 10, and 11 isolated from a hepatic abscess in sheep, prescapular LN in sheep, and mammary LN in sheep, respectively, were clustered together (C2) with a dissimilarity of 4%. Isolates No. 5 and 7 isolated from retropharyngeal LN in sheep and submandibular LN in goats, 0.05 mg/mL, were also shown in the same cluster with a dissimilarity of 1%; and isolates No. 1, 3, and 4 isolated from prescapular LN in sheep, lung abscess in sheep, and mesenteric LN in sheep, respectively, were clustered in the same group (C4) with a dissimilarity of 7%. The two clusters (C1 and C2) differ from the other two clusters (C3 and C4) by approximately 25% as shown in Figure 3 and Figure 4.

### 3.2. Gross Pathology

**Figure 5:**
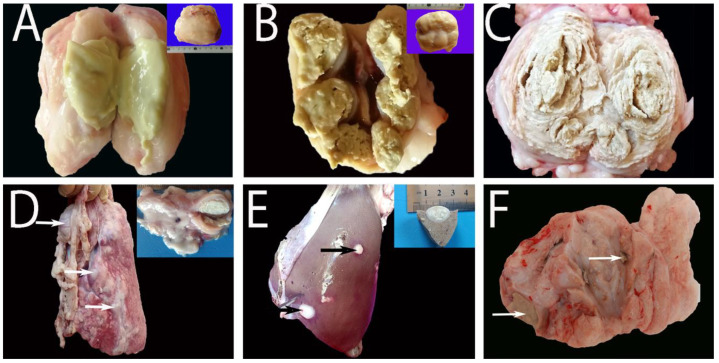
Gross lesions of Sheep naturally infected with *C. pseudotuberculosis* showing (**A**) marked enlargement of the prescapular lymph node with a thick, creamy, green pus. (**B**) Marked enlargement of the prescapular lymph node by multiple encapsulated abscesses with a central light green inspissated core surrounded by a dense fibrous capsule. (**C**) Concentric lamellated abscess in the prescapular lymph node. (**D**) Abscesses of variable sizes were randomly distributed throughout the lung parenchyma (arrows) and mediastinal lymph node (inset). (**E**) Multiple nodules within hepatic parenchyma (arrows) (a formalin-fixed specimen(inset)) contained a central necrotic calcified core surrounded by a dense fibrous capsule. (**F**) Multiple abscesses within the mammary gland (MG) are present (arrows).

Examined lymph nodes demonstrated marked enlargement (3–10 cm) with either thick creamy green pus (Figure 5A) or multiple abscesses consisting of a central caseated core surrounded by dense fibrous connective tissue capsules (Figure 5B). Some lymph nodes exhibited concentric lamellated layers or an onion-like appearance in cross-sections, which is pathognomonic pattern of CLA (Figure 5C). Similar lesions of variable-sized abscesses were randomly distributed throughout numerous internal lymph nodes as mediastinal LN and other parts of the body, especially in the lung (Figure 5D), liver (Figure 5E), and MG (Figure 5F) in sheep and goat carcasses.

### 3.3. Histopathological Results

**Figure 6:**
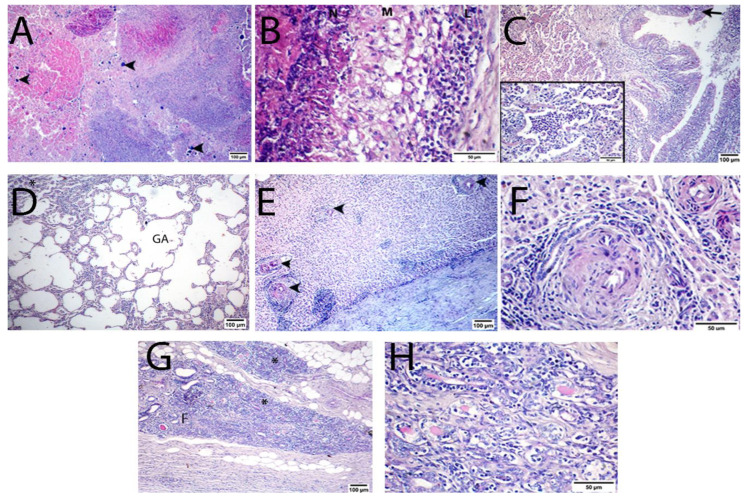
H&E-stained photomicrograph of corynebacterial lesions in sheep. (**A**) Center of corynebacterial pyogranuloma in liver displaying caseous necrotic mass, bacterial colonies, multifocal calcification (arrowheads), pus cells, and intense mononuclear cells infiltration. (**B**) The periphery of a corynebacterial pyogranuloma in the liver displaying the organization of chronic inflammatory cells in three layers: a layer of the pyogenic membrane (N) surrounding necrotic material, a macrophage layer (M), and a lymphocytic layer (L). (**C**) Lung section demonstrating suppurative bronchopneumonia with epithelial necrosis and desquamation (arrow), with prominent neutrophilic infiltration within alveoli visible in the inset. (**D**) Lung section demonstrating the development of a giant alveolus (GA) surrounded by atelectatic alveoli (*). (**E**) A hepatic section demonstrating multiple foci of portal fibrosis, ductular reaction, and neutrophilic and lymphocytic infiltration (arrowheads). (**F**) A hepatic section demonstrating interface hepatitis with separation of hepatic plates, vasculitis, portal fibrosis, and formation of newly formed bile ductules. (**G**,**H**) Mammary section showing diffuse and complete acinar destruction, intraductal and interstitial mononuclear infiltrates (*) and interstitial fibroplasia (**F**). (**A**,**C**–**E**,**G**) scale bar = 100 µm, (**B**,**F**, inset **C**,**F**) scale bar = 50 µm.

**Figure 7:**
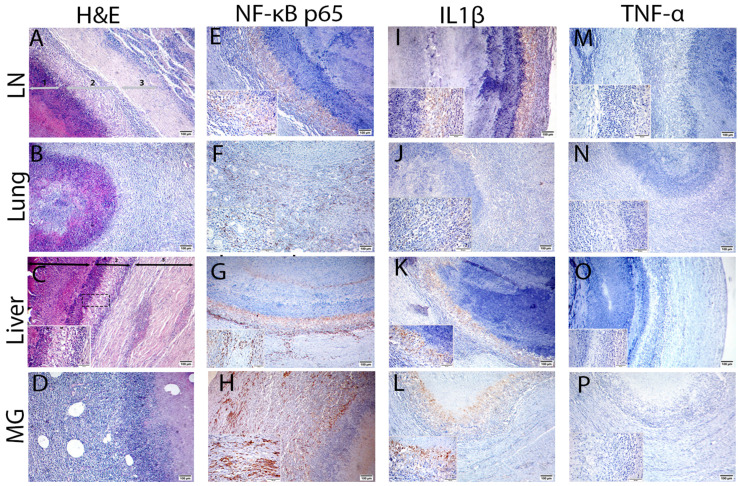
Histopathological and immunohistochemical staining of NF-κB p65/IL1/TNF for ovine corynebacterial pyogranuloma in the prescapular lymph node (LN), lung, liver, and mammary gland (MG). (**A**–**D**) H and E-stained pyogranulomatous lesion exhibiting a typical pattern of CLA consists of a central necrotic core (1) surrounded by a peripheral mantle of chronic inflammatory cells (neutrophils, macrophages, and lymphocytes) (2) then encapsulated by a fibrous capsule infiltrated by lymphocytes (3). (**E**–**H**) NF-κB p65 protein immunostaining showing marked brown immunoexpression in all tissues, particularly the liver and MG. (**I**–**L**) IL1β protein immunostaining showing marked brown immunoexpression to be highly expressed in LN, liver, and MG and moderately expressed in the lung. (**M**–**P**) Immunostaining for TNF protein reveals negative expression in all tissues. All images scale bar = 100 µm, (except insets) scale bar = 50 µm.

Typical suppurative or necrotizing granulomas that compress the surrounding tissue were observed via microscopy. The center of an abscess consists of a necrotic core of liquefied or caseated–calcified material that is primarily infiltrated with degenerated polymorphonuclear (PMN) cells (Figure 6A) and bordered by a thin layer of living polymorphonuclear neutrophils. This is surrounded by a peripheral mantle of active immature fibrosis infiltrated by chronic inflammatory cells (macrophages, epithelioid cells, lymphocytes, and plasma cells) (Figure 6B) and encapsulated by a thick layer of mature fibrous connective tissue capsule composed of well-organized fibroblasts infiltrated by some inflammatory cells such as macrophages and lymphocytes. This pyogranulomatous lesion was found in the LNs, lung, liver, and MG tissues (Figure 7A–D, respectively). Moreover, LNs showed marked lymphoid hyperplasia while the pulmonary parenchyma demonstrated congestion of interalveolar blood capillaries, alveoli, and the bronchial tree, which were filled with exudate necrotic cellular debris, neutrophils, and alveolar macrophages (Figure 6C). Furthermore, pulmonary emphysema with giant alveolus formation and atelectasis were observed (Figure 6D). Moreover, liver sections demonstrated interface hepatitis, dissociation of hepatic plates, hepatocellular degeneration, and necrosis. In contrast, portal areas demonstrated congestion, biliary hyperplasia with newly formed bile ductules (ductular reaction), moderate to severe infiltration of PMN, mononuclear inflammatory cells, vasculitis, and fibroplasia (Figure 6E,F). In addition, mammary tissue demonstrated diffuse and severe inflammatory infiltrates, acinar and ductular destruction, and interstitial fibroplasia (Figure 6G,H).

### 3.4. Immunohistochemical (IHC) Evaluation of NF-κB p65, IL1β and TNF Proteins

IHC evaluation of the inflammatory NF-κB p65 protein in LN, lung, liver, and MG abscesses revealed strong positive brown immunostaining at various layers, especially in the liver and MG pyogranulomas (Figure 7E–H). While IHC analysis for IL1β protein revealed marked positive brown immunoexpression in LNs, liver, and MG abscess, moderate positive brown expression was observed in pulmonary pyogranuloma (Figure 7I–L). Moreover, no immunostained brown cells for TNF protein were detected in LNs, lung, liver, and MG pyogranulomas (Figure 7M–P).

### 3.5. Pathogenicity Test

**Figure 8:**
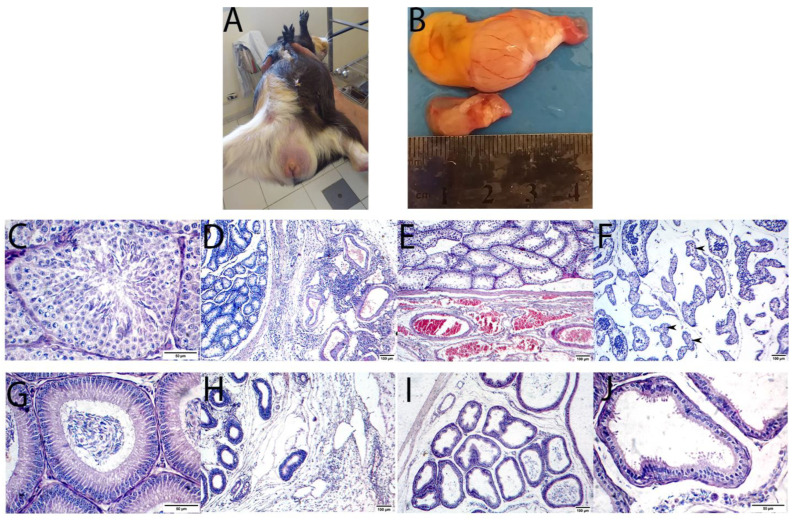
Gross and histopathologic examination of Guinea pigs’ testes and epididymis. (**A**) *C. pseudotuberculosis* inoculated Guinea pigs showing a positive Strauss reaction. (**B**) Testis from positive Guinea pigs showing severe testicular enlargement with hyperemic blood vessels compared with the control animal. (**C**) Testis from the control group displays normal seminiferous tubule and interstitial tissue histoarchitecture. (**D**–**F**) Testes from positive Guinea pigs D and E demonstrating congested and hyperemic vessels and marked mononuclear inflammatory infiltrates in tunica albuginea with azoospermia and degenerated germinal epithelium, while F demonstrates marked interstitial edema, sloughing of spermatogonia cells, and buckling of seminiferous tubule membrane (arrowheads). (**G**) Epididymis from the control group exhibiting normal histoarchitecture. (**H**–**J**) Epididymis of positive Guinea pigs; H demonstrates diffuse infiltration of mononuclear infiltrates in tunica albuginea, whereas I and J demonstrate a complete absence of spermatozoa in lumina along with degenerated ductal epithelial cells. (**D**–**F**,**H**,**I**) scale bar = 100 µm, (**C**,**G**,**J**) scale bar = 50 µm.

Grossly, most injected Guinea pigs exhibited a positive Strauss reaction, and the microorganism was successfully isolated from all positive animals. The testes and epididymis of the control group exhibited a normal gross appearance, whereas the inoculated Guinea pigs exhibited severe testicular swelling with hyperemic blood vessels and an enlarged, firm, yellow epididymis (Figure 8A,B).

Microscopically, testicles of the control group have normal histoarchitecture of seminiferous tubules, interstitial connective tissue, and complete spermatogenesis (Figure 8C). While the testicles of the injected Guinea pigs exhibited periorchitis and periepididymitis, the testicles of the uninoculated Guinea pigs were as seen in (Figure 8D,H). Tunica albuginea and rete testes were infiltrated heavily with mononuclear inflammatory cells. Most seminiferous tubules in the testicles were devoid of spermatids and spermatozoa, indicating moderate to severe testicular degeneration. In moderate testicular degeneration, there was vacuolation, necrosis, and sloughing of spermatogonia cells, along with vacuolation of Sertoli cells, congestion of interstitial blood vessels (Figure 8E), and edema, in addition to the membrane of seminiferous tubules becoming thickened and buckled as a result of its collapse and shrinkage (Figure 8F). In severe cases of testicular degeneration, tubules lacked spermatogonia and spermatids and were lined by vacuolated Sertoli cells. The histoarchitecture of epididymal sections from the control group was normal (Figure 8G). Some epididymal samples contained vacuolated ductal epithelial cells and sloughed germinal epithelium with few or no spermatozoa in their lumina (Figure 8I,J). Observed were also interstitial edema, congestion, and fibrosis with infiltration of mononuclear cells.

## 4. Discussion

Biochemical test results for identifying *Corynebacterium pseudotuberculosis*, excluding nitrate-negative bacteria, were variable, impractical, and less informative than the microscopical image, especially when such coccoid and filamentous rods were shown infrequently, which hindered its applications in routine bacteriology [5,8,34]. The molecular identification with primers targeting the 16S rRNA genes of *C. pseudotuberculosis* yielded rapid and precise results. Nonetheless, the pathogenicity test in male Guinea pigs (Strauss reaction) and pathological characterization cannot be disregarded; they rank second after molecular and serotyping and indicate that the biovar (sertype1. Nitrate-negative) [4,6] is the agent responsible for CLA in sheep and goats. Intraperitoneal inoculation of *C. pseudotuberculosis* in male Guinea pigs can cause acute disease as reported by Hommez et al. [35], distinguishing it from other Corynebacterium species as reported by Pépin et al. [7], Ahmed et al. [36] reported other aspects, and long period for abscess formation (taken up to 10 days with our strain) limited its reliability making PCR the most accurate and rapid method for identification of *C. pseudotuberculosis*.

Similar to other bacteria, an increase in its resistance to antibacterial agents was anticipated, classifying it as a multidrug-resistant (MDR) bacterium, as shown in Table 2 and Figure 2. Additionally, the presence of waxy mycolic acid in its cell wall, which plays a role in its pathogenesis, and mechanical and possibly biochemical protection from the hydrolytic enzymes of phagocytes as a facultative intracellular parasite [1,6], but this is also due to the presence of integron mechanisms such as integron 1, which carried in its cassettes different genes (Figure 3) detected in this investigation. Integrons are mobile DNA elements that, through specific recombination can capture genes, mainly antibiotic-resistant genes [37]. This strain is highly virulent and should be considered (killed and showed orchitis in male Guinea pigs within 2 days). Although the relationship between Corynebacterium’s virulence and antibiotic susceptibility has not been described, it has been observed in *Mycobacterium tuberculosis* [38] and *Klebsiella pneumoniae* [39].

ERIC-PCR is an inexpensive, sensitive, and rapid molecular typing technique requiring no prior genome knowledge [40,41]. A variety of clusters generated in the ERIC-PCR dendrogram showed a similarity of isolates from diverse sheep and goats without any difference between goats and sheep isolates, and geographical area at Matrouh Governorate; inferring that the transmission of this infectious disease from sheep to goats is the likely source of infection, and vice versa. Although the results indicated that different genotypes were within the same serotype 1, the ERIC-PCR clustering analysis can be interpreted as the genetic relationship of the dendrogram from different isolates detected in four clusters. By comparing the ERIC-PCR method to the serotyping method, our results demonstrated that ERIC-PCR is a rapid and reliable method, which furthermore classified the isolates into four clusters (subtypes). Consequently, we believe it can be widely applied in the molecular classification of microbes, particularly *C. pseudotuberculosis* strains [15], making it a useful tool for discrimination. We believe this is due to the endemic nature of the disease, the ineffectiveness of the imported vaccine, and the high tolerance of *C. pseudotuberculosis* to environmental stresses, despite vaccination and the failure of antibacterial treatment. *C. pseudotuberculosis* can survive in the environment for up to 6 months, and CLA is a long-term disease beyond the infected animals that remains in this condition for the duration of their lives, disseminating the agents through purulent discharge of LNs [42]. The knowledge of the genotypic profile of Egyptian strains facilitates the development of an efficient vaccine. Presently, the ERIC-PCR method is employed for molecular epidemiological analysis of human intestinal flora, *Salmonella, Listeria, Haemophilus parasuis*, and *Vibrio cholera* [36].

In our study, gross examination of LNs showed marked enlargement with either thick creamy green pus or concentric lamellated layers (onion-like appearance). Al-Gaabary et al. [43], Singh et al. [44], and Abdulrahman et al. [45] have previously observed similar gross lesions in LNs. Over time, the abscess transforms into a pyogranulomatous lesion in which the purulent material becomes caseated and calcified and is surrounded by a dense fibrous capsule, with active recruitment and migration of leukocytes into the lesion via the collagen layer. Due to its unusual outer lipid structure, *C. pseudotuberculosis* is not destroyed by phagocytosis. It continues to multiply within phagolysosomes, eventually leading to the death of phagocytes [46]. The epithelioid and fibrous reactive layers undergo necrosis as the lesion grows, with the epithelioid layer dying first. Then, outside of this fibrous layer, new reactive layers form, resulting in concentric lamellated lesions resembling an onion ring in cross-section, which is pathognomonic for CLA in small ruminants [10]. Therefore, granulomas are regarded as a protective mechanism restricting bacterial dissemination and metastasis to healthy tissues and stimulating the development of an effective immune response against this pathogen [47]. Similar microscopic findings were observed in our study and in previous research [44,48,49,50]. Notably, the position of activated macrophages between the necrotic core and the lymphocytic layer is consistent with their role as antigen-presenting cells and effector cells against this pathogen [7]. In our study, we also observed lymphoid hyperplasia in lymph nodes, suppurative bronchopneumonia in the lung, massive hepatocellular necrosis and interface hepatitis with portal fibrosis, vasculitis, and ductular reaction in the liver, and complete acinar destruction with intraductal and interstitial mononuclear infiltrates in mammary tissue. Consistent with Sonawane et al. [51], Singh et al. [44], and Umer et al. [52], respectively.

Male Guinea pigs that were injected exhibited a positive Strauss reaction and severe testicular enlargement, while histopathology revealed periorchitis and periepididymitis with testicular degeneration and oligo to azoospermia in the majority of seminiferous tubules. *C. pseudotuberculosis* is spread to the testes and resides within macrophages, resulting in severe tissue destruction [53]. These findings parallel lesions previously described by Gaabary et al. [43] and Umer et al. [52].

Macrophages are most predominant cells in response to many bacterial infections, particularly CLA pyogranulomas. Interferon-γ (IFN-γ) from peripheral leukocytes after *C. pseudotuberculosis* infection activates polarization of activated macrophage to M1 macrophage that is responsible to produce a variety of proinflammatory cytokines, such as IL-1β, and TNF [54]. Unlike TNF, IL-1β is not directly secreted and persists as an inactive precursor protein, pro-IL-1β, which requires inflammasome assembly and caspase-1 activation for maturation [54]. The production of these proinflammatory cytokines in response to *C. pseudotuberculosis* antigen leading to chemotaxis of other inflammatory cells mainly macrophages and lymphocytes to the site of inflammation. IL-1β has many roles which consider a leukocytic pyrogen that induces T activation and B cell differentiation, which is a crucial step in developing a cellular immune response against this pathogen. Additionally, IL-1β induces the release of other cytokines as TNF and IL-2 [55,56]. These mediators’ signaling cascades begin with the translocation of NF-κB to the nucleus for the transcription of these mediators [54].

In this study, IHC analysis for the NF-κB/p65 protein revealed marked immunoexpression in the lymph node, lung, liver, and muscle, well-developed granulomas. LN, liver, and MG pyogranuloma demonstrated strong positive brown expression, whereas pulmonary abscess demonstrated moderate positive brown immunoexpression. To the best of our knowledge, we are the first to document the immune expression of both NF-κB and IL-1β in naturally infected animals with CLA. Othman et al. [57] illustrated that IL-1β was the first cytokine at the early stage of CLA. Moreover, Jesse et al. [58] showed that the marked increment in IL-1β serum levels were correlated with the detrimental pathological lesion in chronic stages after intradermal inoculation of non-pregnant does with *C. pseudotuberculosis*. In addition, negative brown immunostaining for TNF protein was observed in all organs of well-developed granulomas. An in vitro study by Zhou et al. [59] showed that NF-κB signaling pathways are involved in IL-1β and TNF secretion in *C. pseudotuberculosis*-infected macrophages. However, Ellis et al. [59] observed that TNF is locally expressed in ovine pulmonary macrophages in CLA by combining immunohistochemical labeling with in situ hybridization. These discrepancies in TNF expression may be attributed to the stage of infection in which TNF is highly detected in the early stage of infection. An experimental study by Lan et al. [60] showed that the titer of TNF was peaked on the 4th day after *C. pseudotuberculosis* infection in mice and then gradually decreased; therefore, TNF is essential for the development of resistance against this pathogen and is in the early stage. Our results confirm that the NF-κB/p65 signaling pathway is involved in the activation of IL-1β in well-developed CLA pyogranulomas. Therefore, additional research is required to investigate the role NF-κB/p65 in inflammatory response against *C. pseudotuberculosis*.

Study limitations: small sample size of isolates was one of the study limitations. Moreover, antimicrobial susceptibility for Corynebacterium spp should be investigated by the MIC method and not Kirby–Bauer. Moreover, the antibiotic panel used is very limited and does not include two molecules to be considered for primary testing (penicillin for penicillin and vancomycin for glycopeptides as reported again by CLSI M45). Regarding the molecular identification and analysis of *C. pseudotuberculosis*, the use of just one marker (16s) does not confer the proper specificity for *C. pseudotuberculosis* identification, and sequencing is required.

## 5. Conclusions

We provided an important reference for CLA in Matruh Governorate, Egypt, in epidemiological investigation and preliminary etiological analysis. In addition, this study aids in the general development of ERIC-PCR for *C. pseudotuberculosis* genotyping and characterization along with susceptibility to antibacterial in order to select an appropriate antibacterial treatment. In addition, the pathological lesions may aid in the diagnosis of CLA. Furthermore, compared with TNF-α, IL1β, the main proinflammatory cytokine, is more vital in CLA pyogranulomas for the regulation of *C. pseudotuberculosis* infection associated with marked NF-κB immunoexpression in activated macrophages. Therefore, the NF-κB/p65 signaling pathway is involved in the activation of IL1β, and additional immunohistochemical studies are required to determine the various roles of NF-κB/p65 in the inflammatory response within CLA pyogranulomas in order to control this pathogen.

## Figures and Tables

**Table 1:** Primer sequences, target genes, amplicon sizes, and cycling conditions.

Target Gene	Primers Sequences	Amplified Segment (bp)	Primary Denaturation	Amplification (35 Cycles)			Final Extension
				Secondary Denaturation	Annealing	Extension	
*Corynebacterium Pseudotuberculosis* *16S rRNA*	F-ACCGCACTTTAGTGTGTGTG R-TCTCTACGCCGATCTTGTAT	816	94 °C 5 min	94 °C 30 s	58 °C 40 s	72 °C 45 s	72 °C 10 min
Integron1 gene cassette (Int1)	F-CCTCCCGCACGATGATC R-TCCACGCATCGTCAGGC	280	94 °C 5 min	94 °C 30 s	50 °C 30 s	72 °C 30 s	72 °C 7 min

## Data Availability

Not applicable.
